# Image standards in Tissue-Based Diagnosis (Diagnostic Surgical Pathology)

**DOI:** 10.1186/1746-1596-3-17

**Published:** 2008-04-18

**Authors:** Klaus Kayser, Jürgen Görtler, Torsten Goldmann, Ekkehard Vollmer, Peter Hufnagl, Gian Kayser

**Affiliations:** 1 UICC-TPCC, Institute of Pathology, Charite, Berlin, Germany; 2 IBM DeepComputing, Brussels, Belgium; 3 Institute of Pathology, Research Center Borstel, Borstel, Germany; 4 Department of Telemedicine, Institute of Pathology, Charite, Berlin, Germany; 5 Institute of Pathology, University Freiburg, Freiburg, Germany

## Abstract

**Background:**

Progress in automated image analysis, virtual microscopy, hospital information systems, and interdisciplinary data exchange require image standards to be applied in tissue-based diagnosis.

**Aims:**

To describe the theoretical background, practical experiences and comparable solutions in other medical fields to promote image standards applicable for diagnostic pathology.

**Theory and experiences:**

Images used in tissue-based diagnosis present with pathology – specific characteristics. It seems appropriate to discuss their characteristics and potential standardization in relation to the levels of hierarchy in which they appear. All levels can be divided into legal, medical, and technological properties. Standards applied to the *first level* include regulations or aims to be fulfilled. In *legal properties*, they have to regulate features of privacy, image documentation, transmission, and presentation; in *medical properties*, features of disease – image combination, human – diagnostics, automated information extraction, archive retrieval and access; and in *technological properties* features of image acquisition, display, formats, transfer speed, safety, and system dynamics. The next lower *second level *has to implement the prescriptions of the upper one, i.e. describe how they are implemented. *Legal aspects* should demand secure encryption for privacy of all patient related data, image archives that include all images used for diagnostics for a period of 10 years at minimum, accurate annotations of dates and viewing, and precise hardware and software information. *Medical aspects* should demand standardized patients' files such as DICOM 3 or HL 7 including history and previous examinations, information of image display hardware and software, of image resolution and fields of view, of relation between sizes of biological objects and image sizes, and of access to archives and retrieval. *Technological aspects* should deal with image acquisition systems (resolution, colour temperature, focus, brightness, and quality evaluation procedures), display resolution data, implemented image formats, storage, cycle frequency, backup procedures, operation system, and external system accessibility. The lowest *third level* describes the permitted limits and threshold in detail. At present, an applicable standard including all mentioned features does not exist to our knowledge; some aspects can be taken from radiological standards (PACS, DICOM 3); others require specific solutions or are not covered yet.

**Conclusion:**

The progress in virtual microscopy and application of artificial intelligence (AI) in tissue-based diagnosis demands fast preparation and implementation of an internationally acceptable standard. The described hierarchic order as well as analytic investigation in all potentially necessary aspects and details offers an appropriate tool to specifically determine standardized requirements.

## Introduction

Tissue-based diagnosis is considered to be the most accurate and, in addition, inexpensive diagnostic technique in medicine. It is subject to considerable changes in respect to implementation of newly developed technologies. These comprise two main fields, namely molecular biology tools including molecular genetics, as well as digital information acquisition and distribution [1-4]. Telepathology, which is the transfer and viewing of macroscopic and microscopic images at a distance, served as the main promoter in developing the digitalization of histological glass slides and viewing microscopic images on a TV screen [5-10]. It was fully established in the early 1990s and was followed by the construction of specific telemedicine systems such as the iPATH or UICC-TPCC at the beginning of this century [11,12]. At present digitalization of a complete glass slide is commercially available as well as internet – accessible automated measurement systems such as EAMUS™ [13,14].

Diagnosis of radiological images obtained from computerized tomography (CT) or magnetic nuclear resonance (MR) underwent comparable changes too: the viewing of radiological films has been replaced by viewing completely digitized radiological images. These changes have been remarkably promoted by its expense savings over conventional film, its development, and environmental considerations in reducing the pollution induced by film development by-products [15-18]. The technology has been matured, and spatially completely distributed diagnostics are offered that separate the diagnostic work with the patient (CT, MR imaging, etc.) at the hardware localization from viewing the images by radiologists and, in addition, from typing the radiologists' dictations at a different, third, location by secretaries [19,17,20,18]. Adequate standards of image acquisition, interaction with the hospital information system and data documentation have been implemented contemporarily with this development [21]. Picture Archiving and Computation System (PACS) and the DICOM standard have to be mentioned herein [19]. These regulations and internationally accepted standards are nearly missing in diagnostic pathology to our knowledge. There exist recommendations and regulations of laboratory practice which are included in the Code of Federal Regulations (CFR) of the United States of America as well as those mentioned in Good Laboratory Practice (GLP), and veterinary pathologists have stated a toxicologic pathology position paper on pathology image data (regulatory forum). Whether these recommendations can be transferred into human diagnostic pathology still remains an open question.

The same consideration holds true when taking a closer look at the working conditions of radiology and diagnostic pathology: Digital images acquired from histological slides are in use for medical diagnosis in a similar manner compared to radiological images; however, the specific conditions differ at least in image nature (black and white versus colour) and clinical environment (radiological images have to be viewed by clinicians working in different disciplines too, for example by surgeons, whereas the judgement of microscopic images completely remains a domain of surgical pathologists).

In this article we analyze the theoretical background and recent developments of microscopic image analysis and preposition to work out appropriate standards. A basic scheme of three different levels in a hierarchic order each consisting of three different "columns" (legal, medical, technological) is suggested. In addition, we want to provide tools that can serve to successfully implement such standards in the daily practice of virtual microscopy in terms of distributed and interdisciplinary medical information transfer.

### Definition of internal standards, workflow in diagnostic pathology and virtual microscopy

#### Human diagnostics

A standard to be useful in tissue-based diagnosis has to fulfill all conditions that permit an easy and reliable generation, distribution and access to patient – oriented medical information [22,7-29]. In addition, it has to supervise all steps of medical information created in and distributed by an institute of pathology. Thus, the aim is to define prescriptions and their appropriate use to permit individual diagnostics without taking care of specific influences that might be necessary for this process or, on the other hand, might disturb or hinder an effective work.

The starting point of such an information handling and distributing system is the laboratory work that necessarily has to separate the tissue from the patient. The mandatory unequivocal attachment of tissue and patient is commonly performed by using bar codes that permit distributed laboratory work without losing the connection to the focus point, i.e. the patient. Theoretically, different approaches could be performed too, such as unique classification of colour, size, and appearance of the submitted tissue similar to a finger print; DNA analysis of the tissue prior to further processing, etc. The creation of an ID number allows tissue processing, production of glass slides, diagnostics and creation of the final, patient – oriented report, independently of how many and what kind of glass slides, dictations, etc. are involved [22,30-32].

The workflow in an institute of pathology usually follows a time sequence order of tissue identification (creation of ID number), tissue processing and slide production, patient – oriented diagnostics, secretary work, diagnosis submission to the clinician, and financial aspects (reimbursement). The described workflow is not a simple straightforward procedure. In contrast, there might be several feedback mechanisms necessarily included between glass slide production and final diagnosis. They are induced by mandatory additional stains and external information sources. The patient-oriented diagnostics are critical points of external information influence either going straight forward or opening the feedback mechanisms.

The feedback pathways are opened if a) slide quality is poor, b) crude viewing or c) external (clinical) information suggests additional laboratory investigations. As virtual microscopy replaces only the conventional viewing via a microscope the basics of the described work flow remain the same. However, virtual microscopy offers the chance to interact with all feedback mechanisms directly. For example, it can order additional necessary laboratory work prior to the pathologist's diagnosis.

Obviously, the described work flow depends upon thresholds that trigger the described pathways. The triggering thresholds depend upon values that are defined by the proposed first level of standards, namely:

a) Legal: Which quality can be tolerated without suing the pathologist in case of errors?

b) Medical: Which quality and information compound still permit a reliable diagnosis?

c) Technological: How can quality be measured? Which quality can be automated, monitored, and corrected (or replaced)?

It is quite difficult to answer these questions, especially as the term "quality" depends upon the diagnosis under consideration: Simple and frequently stated diagnoses require less "quality" when compared with difficult or rare diagnoses. Thus, the threshold of "good image quality" has to be adjusted to the unknown outcome or the "final and correct diagnosis".

Selecting appropriate or diagnosis – relevant fields of view is an additional task of a diagnostic pathologist. What is a diagnosis – relevant field of view? The easiest answer is to view the complete electronic image and document all fields of view in combination with the chosen magnification, image brightness, and focus.

On the other hand, constant image quality levels can be introduced if the inductive term "diagnosis" is disregarded, and only image measurements are under consideration. To our knowledge, image measurement and quality standards do exist for static quantitative DNA analysis only [33-37]. The standards permit a standard deviation of 3% when measuring the DNA content (integrated optical density) of Feulgen stained nuclei in cytological smears [38,35,28]. These limits can be controlled by the internet accessible server "EUROQUANT" which can work on both static DNA quantification systems and the performance of laboratory Feulgen stains [38].

#### Machine diagnostics

The development of artificial intelligence and its introduction to virtual microscopy will affect the diagnostic work of a pathologist too. The establishment of distributed tissue-based diagnosis networks will probably occur in a Grid environment and will sooner or later implement automated diagnostic procedures [39,13,2-4]. The implementation will certainly require appropriate standards, which again can be attributed to legal, medical and technological necessities.

*Legal aspects* primarily have to deal with protection of the patient's privacy in a distributed electronic network. Secure encryption techniques have been developed; however, the legal limits of quantitative probability measures to break such a system are still missing to our knowledge. For example, the maximum time allowance to work out an individual diagnosis has not been defined. Obligatory quality assurance by (expert) consultation does not exist as well. Archives have to ensure correct encryption of diagnosis for at least ten years in comparison to glass slide archives. DNA characteristics should be encrypted too. Images used for diagnostic aims have to be labelled. The label should indicate that the image served for diagnostic purposes and also include diagnostic specificities such as primary diagnosis, expert consultation, or measurement. These legal standards have to cover the internal work flow of each individual pathology institution.

*Medical aspects* are associated with the diagnostic procedure and related to the applied diagnostic algorithms. The main issues include permitted levels of diagnosis to be automatically stated or the characteristics and number of biological units that are necessary to state a diagnosis, their displayed structures, and the analysis of underlying tissue texture [2,40,41]. These terms direct to terms of tissue screening (cancer versus inflammation versus normal tissue?), or to features such as minimum number of pixels to build a biological unit (for example nucleus, cell, gland, vessel, etc.), or minimum pixel size of the whole image in relation to the tissue texture. Standards of expert consultation and assurance of diagnostic sensitivity and specificity have to take into account the level and difficulty of diagnosis under consideration. It seems to be more appropriate (and practical) to investigate more frequently and more in detail in difficult diagnoses than just to cover general terms such as invasive ductal breast carcinoma. Teaching and continuous education standards of an automated diagnosis system should not be forgotten as probably the future system will be of a flexible, self adjusting and self organization nature [2,3,14].

*Technological aspects* are related to the aims of the system or network. In radiology, the well known image archiving standard PACS (Picture Archiving and Communication Systems or PAC systems) manages the creation, distribution, and archiving of digital images. It can still be considered as the "internal standard" as it has been specifically developed for diagnostic live imaging. It usually consists of image acquisition and archive devices, as well as diagnostic workstations embedded in a network with reasonable bandwidth, and archive device archive/routing software. The bandwidth is important as radiological images usually comprise 10 – 50 Megabytes per study. All modern PAC systems use the DICOM standard and include a network protocol that runs on top of the existing internet standard protocol (TCP/IP).

Some efforts have been made to include or appropriate adjust the PACS and DICOM standard to the needs of diagnostic pathology [42-46,29]. When designed for the needs of virtual microscopy they have to include hardware and software features. Nature and number of included nodes in a network, interconnectivity, computation velocity, backup procedures, information acquisition, distribution, and display have to be defined in appropriately ranges. System reaction in case of data overload must be included as well as boundaries in computation power [47,24,49,46]. The acknowledged standard of such a system should be considered as "internal" and not as "external" or for communicative standards.

### Communicative standards

#### Basics

Progress in diagnostic medicine is mainly related to acquiring, viewing, and distributing images obtained from a broad variety of sources. When introducing digital images into medical diagnostics it was soon recognized that standards are essential if images produced by equipment of company *A *should be compared with those of company *B*, or if images of different origin such as CT, ultrasound, or endoscopy should be "prepared to one case only". For these purposes the so-called DICOM standard has been released in the late 1980s followed by DICOM 3 in 1993. The acronym, DICOM stands for *Diagnostic Images and Communication in Medicine*. DICOM 3.0 is now universally respected and acknowledged as international standard. DICOM standardized images contain much more information than the basic image. They possess "standardized" headers that permit identifying the patient, details of image acquisition and processing (for example, whether the image is unaltered or "enhanced") [50]. Several working groups deal with the application of DICOM standards in specific medical disciplines, and the working group 26 (WG 26) has been established to propose a common appropriate model for combining both HL 7 and DICOM 3 in tissue-based diagnosis [43].

#### Legal aspects

They include those stated for internal standardization such as patient's privacy, labelling of transferred images, date and time schedules as well as notification of image properties in order to avoid mistakes.

#### Medical aspects

In addition to the internal standards, reasons for communication and transfer of medically important information are required. The reasons of specific area selection should be documented when submitting still images. The number of transmitted still images, the chosen magnification, and specific stains should be noted. The standards should be adjusted to the clinical diagnosis. In general, an average number of 4–6 images, acquired by at least 2 different magnifications, can be considered as baseline [3,25]. Images acquired from H & E stained slides are essential, those from adequate immunohistochemical procedures usually helpful [3,25]. These recommendations derive from telepathology experiences [51,6,53,49,46]. Tissue micro arrays (TMA) should be included in these recommendations despite they frequently serve for scientific investigations only [54,55].

#### Technological aspects

Recommendations for adequate standards are related to data security, transfer speed, reliability of data transmission, and stability of line connections, and aspects of image compression [47,56-58,46]. In addition, technological standards that regulate adequate signal release and receipt with correct understanding such as IEEE 802.11, a standard family for wireless local area network (WLAN), are essential. The general implementation of the internet and cellular phones has extensively promoted and implemented general available communication protocols and standards [59]. However, they need to be adjusted to the specific conditions of tissue-based diagnosis.

### Considerations image acquisition, and graphical presentation of virtual slides

It is a primary requirement that pathologists should be provided with sufficiently high-quality images for carrying out effective diagnoses.

Histological images are commonly acquired by attaching several frames of an image, and to automatically compose a wide field of view and high resolution image from these frames. The final image consists of several attached digital image mosaics (patchwork). The jagged images then need smoothness for correct presentation. The generated images or virtual slides are always available as Bitmaps.

The electronic presentation of virtual slides depends upon the image acquisition system, the optical resolution in comparison to the capabilities of the human eye (to be assumed one micron), and the light colour balance in order to display the colours as truly as possible. The pathologist wants to view and manipulate virtual slides in the same way he is handling the light microscope. Therefore, the mandatory image processing is a key point and has to leverage signal-processing capabilities developed in other scientific fields. Afterwards, image archiving and retrieving is more or less a matter of available information technologies.

The necessary image processing is related to signal-processing in general and coordinates features of image acquisition, transfer and display. In addition to specific algorithms, architectures for image coding, filtering, restoration, segmentation, colour reproduction and display, the image size is of major significance.

Image size refers to the physical dimensions of an image, and is usually based upon a fixed number of pixels. Increasing the size of an image decreases its resolution, and decreasing its size increases its resolution. Image resolution refers to the spacing of pixels in an image. The higher the resolution, the more pixels form the image. Higher resolution allows for more detail and subtle colour transitions in an image.

The display of an acquired image (virtual slide) is the tool the pathologist is working with. Its optical resolution is expressed in pixels per square inch; (e.g. 600 dpi dots per inch; a 22 inch screen can present 9 million pixels). The precision and the colour truth of the pixels contribute to the displayed image quality too. Therefore, the size of the screen, the number of pixels the screen can display, the brightness of the screen (measured in Lumen), the contrast ratio (e.g. 2000:1), and the colour depth expressed in bits to represent each colour (e.g. 24 bit RGB) are a matter of technological image standard. In practice, the screen resolution depends on the technology chosen and include SVGA 800*600, or XGA 1024*768, or SXGA 1280*1024 as the most recent technology available.

### Level of standards to be applied in tissue-based diagnosis

Standards should permit constancy in work performance as well as in information release and receipt [22,23,60,15,6]. In addition, they should assure a certain quality level. Introducing standards in tissue-based diagnosis has to overcome several constraints. These include conservative behaviour of pathologists, fears of additional costs, regulations by external institutions, and commonly missing technological knowledge. We therefore propose three hierarchical levels of standards in order to demonstrate the nature and potential effects in the daily work of a surgical pathologist. The levels are listed in table 1.

**Table 1:** Proposed levels of standards in tissue-based diagnosis

**Level**	**Legal aspects**	**Medical aspects**	**Technological aspects**
**1 (Highest, aims: what to do?)**	privacy, image documentation, transmission, presentation	disease – image combination, human, automated diagnosis, archive	Image display, formats, transfer speed, safety, system dynamics
**2 (Intermediate, implementation: how to do?)**	encryption of privacy data, 10 years image archives, annotations of dates and viewing, precise hardware and software information	DICOM 3, HL 7, image resolution, fields of view, of sizes of biological objects and image access to archives and retrieval	PACS, image acquisition systems, image correction procedures, display resolution, image formats, storage, cycle frequency, backup procedures, operation system, external system accessibility
**3 (Lowest, actual standard data: to do with what?)**	Maximum error rate, individual prepositions, institutional characteristics.	Diagnosis frequencies, dates of continuous education, quality control.	Control check of devices and used systems, device and software maintenance.

The first level (level 1) provides information about the included standardization aspects. It "triggers" the next (second) level, and is comparable to the labels of an archive system. Level two generates the necessary items to be standardized, and level three contains the actual data (permitted thresholds or limits). The "columns" at each level are independent from each other. Thus, technological development can be included without changing the legal or medical positions, and vice versa.

Grading standards into a proposed level ensures flexibility and introduction of new components as well as concrete implementation of standards. For example, DICOM 3 handles the general performance of image acquisition, transfer and display in terms of basic rules [50]. However, it cannot provide actual understanding of incorporated devices in all conditions; the local specificities have to be adjusted individually.

The remaining workload in order to establish a wide spread and detailed medical standard can be judged from table 2. An univocal disease classification is the prerequisite for any additional steps in expanding medical standards. The WHO classification of tumours has to be mentioned here, as well as the disease and cancer classification schemes ICD-O and SNO-MED. However, no investigations have been undertaken to refer these standards to disease-specific image characteristics to our knowledge. Such standards would permit reproducible analysis of virtual slides in relation to the stated diagnosis and, in addition, quantify diagnostic accuracy and difficulty.

**Table 2:** A survey of medical standards (level 2 and level 3) suggested or already implemented in tissue-based diagnosis

**Medical Standard (level 2)**	**Criteria (level 3)**	**International/national Action**
Disease order [41]	Hierarchic, 1 – 10	Proposed
Formal disease classification	ICD-O, SNO-MED	Accepted
Disease/human classification	Board discussion	Accepted for tumours (WHO classification)
Disease frequency	Not evaluated, probably >2 per month	Open
Mandatory expert consultation	Rare cancer, lymphoma	Proposed
Mandatory quality assurance	Breast cancer, Hodgkin's disease	Accepted
Image handling, exchange by DICOM 3, HL 7	General & individual regulations	Proposed
HIS, RIS communication by DICOM 3, HL 7	General & individual regulations	Proposed
Expert communication guidelines	4 – 8 images/case, <2 different magnifications/case,	Proposed

*Virtual microscopy:*		

Diagnosis/image properties	>50 pixels/object, >40 objects/image, >256 × 256 image size	Proposed
Disease/image features	Disease – associated texture, object, structure parameters	Open
Image quality measure	Gray value deviation, image standardization (shading, gray value range, gradient)	Open
Screening/image size	Texture features: crude screening Object/structure features: classical diagnosis (H&E)	Open
Diagnosis/colour space	RGB space – classical diagnosis. HSI space – prognosis associated diagnosis, cancer scores (hormone receptor, Gleason, etc.)	Open

The hierarchic disease order as proposed by Mireskandari et al. [41] grades the "distance" of an original stated diagnosis from that stated by the consulted expert. The proposed error grading is shown in table 3.

**Table 3:** Proposed error grading of potential medical differences between primary and expert diagnosis (according to [41].

**Grade**	**Error in**	**Differences**
0	*Rejection or No answer*	
1	*Organ tissue*	Normal <> Abnormal
2	*Abnormal tissue*	Inflammatory <> Neoplastic
3	*Lesion*	Acute <> Chronic; Benign <> Malignant
4	*Cell type*	Sarcoma <> Carcinoma, Tumor <> Inflammation; Non- <> Invasive
5	*Specificities*	Tbc; Fibroma <> Myoma; Adeno <> Squamous
6	*Severity, Grading*	low <> high
7	*Origin, bacteria*	Tbc <> Brucellosis; Primary <> Metastasis
8	*Prognosis*	Good <> Fair <> Poor
9	*Therapy, follow up*	No need <> yes
10	*Identical diagnosis*	*minor differences in differential diagnosis*

Obviously, it is related to the clinical significance of medical disagreement and fulfils the requirements of a standard measure in abbreviating medical diagnosis. Formal disease nomenclature should be distinguished from the classification of "disease meaning" which has to be standardized by intensive board discussions of the corresponding specialists. The embedding of image acquisition, handling, distribution, and display properties such as DICOM 3, HL 7 try to combine medical demands with technical tasks [43,29]. They are tools that have successfully replaced the conventional work flow in live imaging [15,21,17,18]. The widely unknown expert communication guidelines have been derived from telepathology experiences [6,2,3]. According to the data of open telepathology systems such as iPATH or the UICC-TPCC, most requesting pathologists fulfill these requirements without knowing them. The mentioned standards to be applied for virtual microscopy still wait for further development and investigation. One should be aware of these items when undertaking efforts to implement compartments of digital pathology. Prerequisites to establish good performance in virtual microscopy include the extraction of object or texture related features from microscopic images. They can ensure accurate diagnoses and avoid remarkable errors [40].

## Discussion

Anatomical surgical pathology or tissue-based diagnosis is on its way to remarkably changing its working conditions [61,39,63,29]. Viewing or analyzing microscopic images will still remain the domain of diagnostic surgical pathology; however, its technological procedures are influenced by innovative developments of visual information acquisition and distribution technology. Surgical pathology presents with highly specialized visual medical diagnostics that includes low costs and minimum error rates. It possesses some similarities to live imaging techniques such as CT, MR or ultrasound examinations. On the other hand, it also remarkably differs from radiology. The differences include formal and medical image properties, interactions with other medical disciplines such as surgery or oncology, and specific laboratory technology. Despite these differences, efforts have been undertaken to implement aspects of standardization developed in radiology to diagnostic pathology [43,29]. These efforts are based upon the well known DICOM 3 and PACS standards, and promoted by experiences obtained from telepathology, virtual microscopy, and Grid technology [39]. These technologies push the work flow of surgical pathology to be integrated into so-called hospital information systems (HIS), often in combination with a radiology information system (RIS). The specific working conditions of tissue-based diagnosis are difficult to understand for HIS and RIS specialists, despite the fact that nearly all institutes of pathology use pathology-specific documentation and archiving systems. This does not neglect the need for automated patient registration data and tissue data transfer from the HIS to the RIS or to apply HL7, an acronym for Health Language 7 for these purposes. HL 7, a standard text communication between different documentation systems, can be considered to be the DICOM equivalent for these purposes. It includes, in addition, a personal patient identification number which can be used without changes to further identify the patient's tissue and derived glass slides.

It seems appropriate to "grade" necessary or proposed standards into hierarchic levels. These levels serve different purposes: The *first level *describes the aims and proposed actions of the standard. Three different items can be distinguished in relation to the aim: legal, medical and technological aspects. Legal items include the patient's privacy as well as documentation prescriptions, image labeling, or display performance. They should protect the pathologist against inadequate diagnostic procedures, such as diagnostic statements obtained from inadequate images.

The term "medical standards" includes disease – related issues that require specific attention in tissue-based diagnostics. For example, the diagnosis "carcinoma of the lung" can be stated even when the quality of viewed glass slides is not optimal in the majority of cases, in contrast to the diagnosis of "usual interstitial pneumonia", which requires high glass slide quality, specific knowledge by the pathologist, and detailed clinical information [22,64,3,4,66]. These specific medical conditions become even more important when virtual microscopy combined with artificial intelligence are applied.

The last aspect is of a technological nature. It includes the technical prepositions to permit accurate diagnostic performance as described by legal and medical aims. The resolution of display units has to be adjusted to the requirements of medical performance as well as computation speed and image transfer time.

The *second level *regulates the implementation of the aims formulated in the first level. It contains the usual descriptors such as DICOM 3 or PACS. Most of the authors who work on standard implementation specify the regulations given at this level [43,50,66]. There are several working groups analyzing the benefits and potential improvements of DICOM 3 [50]; others try to enhance archive systems by changing details of PACS [30,67,44].

The *lowest (third) level *includes the actual limits of accuracy and allowed error rates. Medical conditions such as minimum number of diagnosed specific diseases that are mandatory to further diagnose the disease without expert consultation are included as well. Technically, the actual data of communicative and other digital standards are noted within this level.

From the theoretical point of view, standards serve for a) internal institutional and b) inter-institutional communication. They ensure a correct "understanding" between different embedded devices and act, therefore, like a common language [6]. Internal institutional application provides a better self organization and is, in addition, an appropriate tool to assure certain quality levels.

In aggregate, implementation of legal, medical, and technological standards into routine tissue-based diagnosis is an important issue in further developing this medical discipline. It still requires clearly defined statements for what to do and for which purpose, statements that could be classified, tested, and implemented according to the proposed classification levels. The introduction of virtual microscopy as well as combined AI to significantly support the efficiency, accuracy, and reputation of the pathologists' work demands new strategies in individual and communicative actions and performance. We do hope that this article will significantly promote pathologists' understanding of the forthcoming changes, and that they might steer and not just react to the unavoidable, however also promising the so-called globalization or digital world of future medicine.

## Competing interests

The authors declare that they have no competing interests.

## Authors' contributions

All authors read and approved the final manuscript.
